# Publication bias is bad for science if not necessarily scientists

**DOI:** 10.1098/rsos.240688

**Published:** 2025-04-30

**Authors:** Remco Heesen, Liam Kofi Bright

**Affiliations:** ^1^ Department of Philosophy, Logic and Scientific Method, The London School of Economics and Political Science, London, UK

**Keywords:** publication bias, filedrawer effect, preregistration, philosophy of statistics, replication crisis

## Abstract

It might seem obvious that the scientific process should not be biased. We strive for reliable inference, and systematically skewing the results of inquiry apparently conflicts with this. Publication bias—which involves only publishing certain types of results—seems particularly troubling and has been blamed for the replication crisis. While we ultimately agree, there are considerable nuances to take into account. Using a Bayesian model of scientific reasoning we show that a scientist who is aware of publication bias can (theoretically) interpret the published literature so as to avoid acquiring biased beliefs. Moreover, in some highly specific circumstances she might prefer not to bother with policies designed to mitigate or reduce the presence of publication bias—it would impose a cost in time or effort that she would not see any benefit in paying. However, we also argue that science as a social endeavour is made worse off by publication bias. This is because the social benefits of science are largely secured via go-between agents, various non-experts who nonetheless need to make use of or convey the results of scientific inquiry if its fruits are to be enjoyed by society at large. These are unlikely to be well-informed enough to account for publication bias appropriately. As such, we conclude, the costs of having to implement policies like mandatory pre-registration are worth imposing on scientists, even if they would perhaps not view these costs as worth paying for their own sake. The benefits are reaped by the go-between agents, and we argue that their perspective is quite properly favoured when deciding how to govern scientific institutions.

## 1. Introduction

*Publication bias* occurs when the outcome of an experiment or research study influences the decision whether to publish it (this phenomenon is also known as the filedrawer effect). Most prominently, ‘null results’ (i.e. studies that fail to reject some null hypothesis of no effect at a relevant level of statistical significance) are almost entirely absent from the literature in psychology, medicine and other fields [1,2]. The presence of publication bias is often taken to detract from the overall reliability of science [3], and has been blamed for the replication crisis in psychology as well as various other fields ([4], §2.2). Many people, including ourselves, think that it ought therefore be the target of ameliorative policy.

However, the matter is not straightforward. There is debate about whether publication bias presents a problem for the epistemic reliability of scientific reasoning (see [5–7]), i.e. about whether scientists can learn effectively from a literature affected by publication bias. Further, popular policies to address publication bias have been cast into doubt as not worth the cost or effort [8]. So to make the case that publication bias is worth addressing through policy one must show not just that publication bias is epistemically harmful, but also that the value of the information gained justifies any associated costs. An adequate resolution to this debate must therefore consider not only the statistical issues at stake, but also take a stance on the role of science in society.

What one means by ‘successful *anti-publication bias policy*’ depends crucially on what one takes the problem or problems created by publication bias to be. For our purposes, we assume the main issue created by publication bias is that people who need to make use of scientific information do not have access to an accurate picture of the state of the science based on the published literature (to foreshadow, we think it is crucial who exactly one takes the relevant ‘people’ here to be; see §4). A successful anti-publication bias policy would thus be some behavioural or institutional arrangement that puts those people in a position to know what experiments have been done and what they found regardless of what those experiments concluded. For example, there is presently a push for the preregistration of studies [9–11]. The broad idea is that ‘[i]n pre-registration, researchers describe their hypotheses, methods, and analyses before a piece of research is conducted, in a way that can be externally verified’ ([12], p. 2). This practice is gaining growing support among social scientists because, among other things, ‘preregistration of studies can reduce the impact of publication bias—particularly the prioritization of publishing positive over negative results—by making all planned studies discoverable whether or not they are ultimately published’ ([10, p. 816]; see also [9, p. 2602]). As this practice spreads, we can start to measure its effectiveness [13]. However, preregistration is not the only possible form of anti-publication bias policy (nor is addressing publication bias the only benefit of preregistration). One might also create journals that specialize in and incentivize publishing negative results, or evaluate study designs and agree on publication before results are in.

We evaluate the question whether there is any need for anti-publication bias policy by considering the strongest case that can be made against it. The reason for this approach is that it is very difficult to make all-things-considered judgements on the social costs and benefits of one way of doing science over another. So we take a more indirect approach. We grant a hypothetical sceptic very favourable assumptions to their case; in particular, we presuppose that scientists are ideally capable of identifying and correcting for the effects of publication bias. We then consider the question: if (counterfactually) scientists were this skilled at correcting for publication bias, would we still need to implement anti-publication bias policy? We argue that they would, thus suggesting that in real life (where presumably scientists are not so skilled) there is an even stronger case for anti-publication bias policy.

More specifically, we respond to one set of arguments (as for instance in [8]) that revolve around the claim that preregistration considered as an anti-publication bias strategy is not worth it, because what we need to do is ensure scientists produce and reason in line with better and more clearly articulated theories. These theories are cognitive tools that enhance the capacities of scientists to reason sensibly about the world, and can also guide them in forming more sophisticated expectations about what sort of underlying processes give rise to the observed pattern of results in a scientific literature. If scientists collectively improved their reasoning in this way, preregistration would simply be unnecessary. In the absence of this kind of more sophisticated reasoning, preregistration is at best a very poor substitute.

The thesis of this paper is that, even given ideal assumptions about scientists’ ability to reason about the impact of publication bias, we would still be better off implementing mandatory pre-registration or some other anti-publication bias policy. In §§2 and 3, we set out to develop a line of argument in the spirit of [8] in the strongest form we can. We introduce and reason with a Bayesian model of a scientist considering the value of information they might gain from learning the results of studies unpublished owing to publication bias. We show that, first, a sophisticated and well-informed scientist would be statistically consistent in their reasoning even where there is publication bias. Second, one can construct situations wherein such a scientist would rather forego the effort involved in gaining the extra information anti-publication bias policy aims to bring out, preferring instead to work with the biased publication record. The assumption that scientists are sophisticated and well-informed Bayesian reasoners thus provides a way of exploring a set of circumstances highly favourable to the opponent of anti-publication bias policy. In line with the argument of [8], we find that where scientists are able to bootstrap their way to such a capacity, or at least approximate it via cognitive aids such as good theories, anti-publication bias policy may be unnecessary.

In §§4 and 5, we turn around and consider how a defender of anti-publication bias policy might respond to these arguments, without disputing the favourable assumptions we have granted to the opponents of such policy. We first argue that science has a broader social role which requires the translation of scientific results by *go-betweens*, people like journalists or civil servants who spread or implement ideas based in scientific research. These go-betweens are unlikely to be sufficiently well-informed to account for publication bias in the sophisticated way we have granted (for the sake of argument) working scientists might be able to. As we show, such go-betweens stand to benefit more from anti-publication bias policy than working scientists.

By undermining what we take to be the strongest argument against anti-publication policy, we take ourselves to have strengthened the case in favour of such policy. However, as we highlight in the conclusion, the work does not end there. The various proposed policies still need to be evaluated on their relative merits, as do the costs of implementing them and potential side effects.

## 2. How does publication bias affect a well-informed scientist?

In this section and the next we ask whether a working scientist whose epistemology and decision-making can be modelled using Bayesian statistical methods would think publication bias harms her.^1^ We begin with the following, rather strong assumption (we abandon this assumption in §4): the scientist knows precisely under what conditions experiments are published. In particular, she knows that publication bias exists (if it does), and she knows under what conditions a study ends up in the filedrawer rather than being published.

To make this idea more concrete, consider the following example, versions of which we will return to throughout. Suppose the scientist wants to learn the mean θ of a normal distribution with known variance. Suppose further that other scientists are gathering relevant data in the form of (identically sized) random samples from this normal distribution. For each such dataset, the sample mean X is a *sufficient statistic* for θ, so we let X represent the data. Writing $\tau^{2}$ for the (known) variance of the sample mean, we have $X|\theta ∼N\;(\theta ,\tau^{2})$, i.e. the sample mean is normally distributed with mean θ and variance $\tau^{2}$.

Now assume that (owing to publication bias) a given dataset is published only if it can be used to reject the null hypothesis that θ=0 (in a standard two-tailed *Z*-test at some pre-specified significance level α). Equivalently, the dataset is published only if the absolute value of the sample mean exceeds some threshold value k>0, i.e. publication is conditional on |X|>k.^2^

Suppose our scientist learns only of data that is published, and that she has no way of knowing whether any other datasets were gathered that failed to be published. As it turns out, a single dataset is published, with sample mean Y=y.

If the scientist naively ignored publication bias, she would treat Y as if it were a random draw from a normal distribution and proceed to update her beliefs using Bayes’ law accordingly.^3^ More precisely: the likelihood function she uses is the likelihood function associated with X rather than Y. If, for example, her prior for θ was a normal distribution with mean μ and variance $\sigma^{2}$, then her posterior for θ would also be a normal distribution, with mean and variance


$$m=\frac{\sigma^{2}}{\sigma^{2}+\tau^{2}}y+\frac{\tau^{2}}{\sigma^{2}+\tau^{2}}\mu \;\text{and}\;s^{2}=\frac{\sigma^{2}\tau^{2}}{\sigma^{2}+\tau^{2}},$$

respectively (this is a textbook application of Bayesian statistics, see [14, p. 179]). Note that the posterior mean is a weighted average of the observation and the prior mean, and the posterior variance is smaller than the prior variance.

Knowing about publication bias, it is better (less naive) to treat Y as following a different distribution than X. In this example, the correct likelihood function for Y is the likelihood of X conditional on the fact that |X|>k, i.e. Y∼X||X|>k.^4^

Assuming the same prior $\theta ∼N(\mu ,\sigma^{2})$, this leads to a more complicated posterior. Compared with the naive posterior, it compensates for the potentially missing data by giving relatively high weight to values of θ near zero.^5^

The approach using the correct distribution for Y is unbiased in the following sense: if Y is sampled repeatedly, the posterior converges in probability to the true value of θ owing to the Bernstein-von Mises theorem ([15], theorem 10.1). This feature (convergence to the true value) is known as *statistical consistency* in the literature. The naive posterior also converges, but not to the true value: its limiting value will be too high if θ is positive, and too low otherwise. In other words, the naive posterior is not statistically consistent.

The example generalizes. Nothing turns on the data or the prior being normally distributed. Moreover, we can substantially weaken the assumption that the only effect of questionable research practices is that all and only statistically significant results are published (whereas in reality, phenomena such as p-hacking and selective reporting further skew the effects and effect sizes reported in the published literature, see [16]), and the assumption that the scientist knows precisely which studies are published. As long as the scientist’s prior incorporates the possibility that the published literature is distorted by publication bias, p-hacking and related phenomena, she can learn the presence and extent of such effects endogenously. In other words, if the scientist starts with a sufficiently open mind about her community’s publication practices, she can learn the presence and extent of distorting practices just by observing published effect sizes ([17,18]; see also [19]).^6^

In typical cases with relatively simple parametric models, these more general approaches will satisfy the assumptions of the Bernstein-von Mises theorem, guaranteeing statistical consistency, i.e. convergence to the true value of the parameter(s).^7^ By contrast, naive approaches that ignore publication bias tend to be biased (inconsistent), as we saw in the example.

As an aside, by positing a true value of the parameter we depart from a strictly subjective Bayesian paradigm. A strict subjectivist would put these large sample results in terms of the agreement that scientists starting with different priors would reach. Scientists using the correct distribution for Y and those using the naive posterior would come to agree among themselves but not with each other. However, in a subjectivist paradigm it is more difficult to make precise the sense in which the former are ‘right’ and the latter ‘wrong’.^8^

The upshot of this discussion is that a sophisticated scientist—one who is sufficiently well-informed about the (possible) presence of publication bias—can account for bias in a principled way. As a result, she has access to a statistically consistent learning method: she need not be systematically misled about what the published results of scientific studies tell her even if publication bias is endemic (and even if it interacts in potentially complicated ways with phenomena like p-hacking).

None of what we have said so far suggests that publication bias is harmless. For all we have said, it could be that even a perfectly informed scientist would be strongly opposed to publication bias, in the sense that she would prefer all studies to be published, despite the fact that she is capable of adjusting her beliefs to the reality of publication bias. This is the question we will address next: should a (well-informed) scientist oppose publication bias?

## 3. Should a working scientist oppose publication bias?

Here is a *prima facie* argument. According to the *principle of total evidence* ([22], §3), when assessing a given question or hypothesis (scientific or otherwise), we should take into account all available information. The presence of publication bias amounts to depriving scientists of information which would otherwise be available to them. Therefore, publication bias should be opposed.

We can make this more precise. Good [23] proves that a scientist using Bayesian decision theory should always use all available evidence. In particular, she would prefer, before making any decisions, to see the data from all studies that have been done on a given scientific question (rather than only the statistically significant ones). Since publication bias prevents this, the scientist should oppose publication bias.

So far, so good for the *prima facie* argument. However, Good’s theorem comes with a caveat:

[I]t pays to take into account further evidence, provided that the cost of collecting and using this evidence, although positive, can be ignored. In particular, we should use all the evidence *already* available, provided that the cost of doing so is negligible ([23, p. 319], original emphasis)

The theorem assumes that ‘collecting and using’ evidence is costless. However, as Good implicitly admits, even evidence that is ‘already available’ comes at a cost: the cost of attending to or absorbing the information. This need not be a financial, but rather an *opportunity cost*: time spent on this evidence is time away from other pursuits.

The opportunity cost of reading and absorbing published data may be low, but not zero. The question is as follows: should a scientist always want to pay the cost to read and absorb data that are not published owing to publication bias? We answer this question in the negative by arguing that no matter how small the cost, there are circumstances where it is not worth paying for the scientist.

The set-up is as before: the scientist is trying to learn the mean θ of a normal distribution, and has some prior for θ.^9^ The sample mean X of each study is normally distributed with $X|\theta ∼N(\theta ,\tau^{2})$. Assume that a number (unknown to the scientist) of such samples has been collected, one of which reaches statistical significance (|X|>k). We compare, first, the publication bias scenario in which the scientist only sees the sample mean of the significant study, and second, the publish everything scenario in which she sees the sample mean of all studies regardless of significance.

We assume the scientist faces some decision problem, such that knowing whether θ is positive or negative is relevant to her decision.^10^ The details of this decision problem turn out not to matter, but they are fixed throughout (i.e. there is no trickery regarding the stakes of the problem at hand). We also fix the cost c of learning and absorbing the value of the sample mean of a single study. We require that c is positive, though c may be arbitrarily small both in absolute terms and relative to the stakes of the decision problem. Note that in the first scenario the scientist only sees one study and hence pays c once, whereas in the second scenario the scientist pays c times the number of studies.

We can now prove that there are circumstances in which the scientist prefers to be in the first scenario rather than the second scenario. The result is stated in terms of the Bayes risk, which is the expected loss associated with the optimal decision (a lower Bayes risk corresponds to a higher expected utility).

**Theorem 1.** *Given* c>0*, if we set the threshold of significance* k *large enough, the Bayes risk in the first scenario is lower than the Bayes risk in the second scenario*.

The proof is adapted from Bayarri & DeGroot ([24], §5), who in turn rely on a theorem due to Blackwell [25,26]. It is included in electronic supplementary material and on the Open Science Framework [27]. The key factors are as follows.

Since the scientist only cares about whether θ is positive or negative, sample means further away from zero are more informative. To see this, think about what a barely positive sample mean tells you as compared with a large positive value. Obviously, positive sample means are more likely to occur if θ is positive than if θ is negative. However a barely positive value still has a decent chance of occurring even if θ is negative, so observing it only makes it slightly more likely that θ is positive. Whereas a large positive value is very unlikely to come up if θ is negative. So a single observation of a very large sample mean will sway the scientist’s posterior strongly in the direction of believing that θ is positive.

Owing to this feature of the problem, a higher threshold k (or equivalently a lower threshold of significance α) guarantees more informative data. In fact, the probability of a wrong decision can be made arbitrarily low by increasing the threshold. So, for k large enough, the risk associated with a wrong decision is smaller than the cost c. This clinches the result: either there are no insignificant sample means (so the Bayes risk for the two scenarios is the same), or the second scenario involves extra observations, which increase the cost by c but cannot lower the risk by more than c.^11^

This provides a proof of possibility that a scientist may sometimes be better off in a world with publication bias. The example almost certainly generalizes (e.g. replace the normal distribution with any symmetric distribution), but as it stands it is close enough to the type of problems that occur in practice to make our point.

It might be objected that in our example, the scientist would most prefer that everything is published while she reads only the statistically significant ones. However, in this third scenario the scientist would have to spend time figuring out which results are statistically significant. Since this comes with an (opportunity) cost, our theorem applies, so there exist values of k large enough such that even this is not worth it.

We emphasize we do not take our example to constitute the typical case, merely a type of case that sometimes occurs in practice. For many statistical problems, more extreme data points are not more informative. Indeed, our argument in the following partially depends on looking at a more typical case and finding that the scientist would have different preferences therein.

The point here is more modest. From §2, we got that the very well-informed scientist we have been considering can in principle learn from data produced in a world with publication bias. From the present section we get that there exist learning problems, perhaps somewhat contrived but not ridiculously so, where the scientist benefits from publication bias. Thus, if we only consider such well-informed scientists, it depends on the context of the learning problem whether publication bias is harmful at all, which puts into question whether (costly) anti-publication bias policy could be worthwhile.

In making what we consider to be the strongest possible case that we need not worry about publication bias, we have helped ourselves to several unrealistic assumptions. We have assumed that our scientist can do sophisticated Bayesian statistical reasoning without making mistakes. We have assumed that all data is collected in good faith and that publication bias takes a very specific form such that all statistically significant findings are published and all insignificant ones are not (whereas in reality the published data is further distorted by p-hacking, selective reporting, and possibly even data fabrication; moreover some significant results might not be published and some insignificant ones published). We have assumed that our scientist knows all this. Also, we have ignored the pressure on academics to publish significant original findings in order to build a career [28–31] and any further distortion to the published literature this causes.

Making our analysis more realistic by incorporating some or all of these factors would probably weaken the case we have just made for not worrying about publication bias. But, since we ultimately disagree with this case anyway, we will not show this in detail. After all, we argue in the next two sections that even if all these unrealistic assumptions about the inner workings of science hold, there would still be a case for anti-publication policy. However, and if they do not then we would want such a policy anyway for independent reasons.

## 4. Science: not for the benefit of scientists

Having first made a case against anti-publication bias policy, we now respond to this case from the perspective of a proponent of such policy.

The foregoing suggests that very well-informed scientists, in particular those with precise knowledge of the presence and nature of publication bias, would not particularly care about anti-publication bias policy, including preregistration insofar as it aims at reducing publication bias or making it more transparent. The ability of such scientists to learn the true distribution of underlying data is not generally reduced by publication bias (§2) and in at least some, admittedly somewhat contrived, cases they actually learn more efficiently in the presence of publication bias (§2) and in at least some, admittedly somewhat contrived, cases they actually learn more efficiently in the presence of publication bias (§3).

Unfortunately, not every consumer of published scientific work is as well-informed as we have been assuming so far. By contrast to the very well-informed scientist of the previous two sections, we can imagine a thoroughly uninformed agent completely naive to the presence of publication bias. Such an agent treats published data as if it were an unbiased sample from the underlying distribution. As already noted in §2, she will learn in a systematically biased way. Unsurprisingly, such an agent stands to benefit more from anti-publication bias policy than the well-informed scientist (as we will illustrate in §5).

This suggests a continuum of informedness along which scientists and other consumers of scientific publications may vary. Most will be somewhere in between the two extremes just sketched: broadly aware of the presence of publication bias but unsure or mistaken to various degrees about the precise nature and extent of publication bias.^12^ Likewise, they are in between the two extremes in how much they benefit from anti-publication bias policy.

This raises the question of who published scientific data is for, and who science more generally is for. If we can establish who the audience is, and how well-informed they can be expected to be, this should give a clue as to how valuable anti-publication bias would be to that audience. To do this, we distinguish between the mediate and immediate aims of scientific research for more on this distinction (see [32], who traces it back to [33]). The immediate aims are those which scientists should be trying to achieve in their research, such as gaining rigorous and precise results, deep or explanatory theories, interesting and illuminating hypotheses, etc. Arguably these can be subsumed under a single immediate aim, e.g. accuracy (for debate, see [32,34,35]). It is at least arguable (and effectively assumed here) that a Bayesian can subsume all these immediate goals into her framework [36].

The mediate aims are the broader purposes of science, the reasons society should invest in science. Perhaps we want to improve public decision making, perhaps we simply value the truth for its own sake, perhaps even we see science as of religious value ([37], §6). We shall take no stance on what in particular the mediate aim of science might be, but we claim that whatever it is it shall require go-between or middle-man agents.

By go-betweens we mean people like science journalists, civil service employees, consultants, lobbyists and other knowledge brokers ([38]; Bortolus *et al*. 2024, Knowledge brokers at the science-policy interface: Insights from biosecurity and environmental management.) who may produce or influence the production of evidence-based policies. Such individuals do not usually directly participate in the scientific community, yet must reason about purported discoveries. This is not a strict dichotomy. Scientists can be go-betweens themselves, e.g. MD-PhDs carrying out medical research, or active scientists who also work as science communicators. Nonetheless, there is a large class of agents outside the scientific community who apply or explain scientific results. These go-betweens’ interpretation of scientific results is often the interpretation which acts as the basis of public information or policy.

Any plausible candidate for a mediate aim of science will require more people than just scientists to be aware of scientific research, thus requiring go-betweens. While arguably scientists’ immediate aim is to have informed and coherent beliefs about their field (for debate, see [32,39,40]), society does not take a special interest in satisfying scientists’ curiosity. Rather, scientific work must be translated into policy guidance or public edification or some other social benefit. Even if the way we socially divide labour were to change quite dramatically, not all of us will be full-time scientists, while policy and politics would still require some input from those with specialized expertise [41]. Thus non-scientists will need to be informed about scientific results [42]. This requires go-betweens, people who take in scientific information without being scientists.

A key assumption in our portrayal of the well-informed scientist was that she knows under what conditions claims are being published. This is a strong assumption even for scientific experts. Its plausibility, if any, derives first from their own lived experience to draw on in understanding the more or less formalized procedures that scientists use to decide whether to publish studies, and second, from their being sufficiently tapped into their epistemic community that they read and appropriately understand papers like Franco *et al.* [43].

However, the vast majority of people do not have such information. Most of us, including most go-betweens, are not scientists and do not keep up with the scientific literature. So there is no reason to believe the go-betweens will satisfy this key assumption. Even after one informs the go-betweens about publication bias, they lack the tacit knowledge gained through active participation in a research community [44], which includes information about the quality and reliability of published work [45]. Whereas if, say, preregistration of studies were the norm, a lot of this information would be much more easily accessible to non-experts [46].

Thus, while we do not claim the distinction between scientists and go-betweens maps onto our continuum of informedness perfectly, we think it reasonable to expect that most go-betweens will tend to be on the less informed side of that continuum. Since, moreover, we have argued that these go-betweens’ consumption of published scientific data plays a key role in securing social benefits from scientific research, it follows that the epistemic situation of a particular group of agents relatively uninformed about publication bias (i.e. the go-betweens) should be of special concern in determining the value of anti-publication bias policy.

## 5. Publication bias is costly for go-betweens

We revisit our case against anti-publication bias policy from the perspective of a naive agent, mostly or fully uninformed about publication bias (as we have argued we should expect most go-betweens to be). Such agents tend to update on published scientific data naively, interpreting the data as an unbiased sample of the underlying distribution. As noted in §2, this way of reasoning is not statistically consistent in a world with publication bias, and can only be safe if all data is available.^13^ Hence, given our focus on the naive agent’s perspective, we should prefer that everything is published.

Now, we noted there are indeed costs to publishing everything. These costs must be accounted for before deciding whether we should prefer that everything is published all things considered. Also we must concede that per theorem 1 there are some scenarios where publication bias is preferable over publishing everything, and this holds even for completely naive agents. So more needs to be said.

However, just because circumstances in which publication bias is beneficial exists, it certainly does not follow that publication bias is harmless or even beneficial in general. To be reassured of this we would need to show that publication bias is harmless or beneficial in typical circumstances. However, in fact the scenario of theorem 1 is quite contrived. For contrast, we now briefly review a slightly altered statistical problem. Apart from the complications introduced by publication bias (and by contrast to the scenario of theorem 1), this is a standard introductory example in statistical decision theory.

As before, the scientist wants to learn θ and has access to one or more studies. Each study is represented by its sample mean X, which follows a normal distribution with mean θ and known variance $\tau^{2}$. The scientist has a prior for θ, which we assume to be normally distributed with mean μ and variance $\sigma^{2}$.

The key change is in the decision problem the scientist faces. We assume the scientist needs to estimate the value of θ under a squared error loss function (whereas before she only cared about the sign of θ). That is, the scientist’s loss is the squared difference between her estimate of θ and its actual value, plus a cost c per sample mean observed.

We draw sample means until we see a statistically significant one (|X|>k). For the numerical results presented below, we assume that significance is determined according to a two-tailed *Z*-test with a false positive rate α=0.05, so $k=z_{\alpha /2}\cdot \tau \approx 1.96\cdot \tau$. We compare the risk (expected loss) for three scenarios:

(i) the well-informed scientist under publication bias. In this scenario, the scientist only observes a single statistically significant sample mean. The scientist is aware of publication bias and uses the correct likelihood function to obtain her posterior for θ;^5^
(ii) the naive scientist (or go-between) under publication bias. The scientist only observes a statistically significant sample mean, but she treats this data point as if it were a random draw from a normal distribution; and
(iii) publish everything. In this scenario, the scientist observes all sample means up to and including the statistically significant one. The scientist correctly treats these as draws from a normal distribution.

In each scenario, the scientist’s estimate for θ is the posterior mean (this is the optimal strategy under squared error loss). Calculating the posterior mean for the well-informed scientist requires integrating the normal distribution function, which has no analytic solution. We thus present some numerical results. However, the following facts can be shown analytically. First, the risk for the well-informed scientist is lower than the risk for the naive scientist. This is because the well-informed scientist follows the optimal strategy for given prior and available data ([14], theorem 12.7), and the naive scientist has the same prior and available data. Second, if the cost vanishes (c=0), then the risk if everything is published is lower than the risk for the well-informed scientist (which is in turn lower than the risk for the naive scientist), by Good’s theorem.

However, as we argued above, the opportunity cost of attending to the data is never zero. Since the scientist always observes exactly one sample mean in the two publication bias scenarios, and the expected number of observations when publishing everything is higher than one, it is clear that if we make the cost c high enough, publishing everything will have a higher risk than the two publication bias scenarios. The question is what happens when c is fairly small but not negligible.

**Figure 1.**
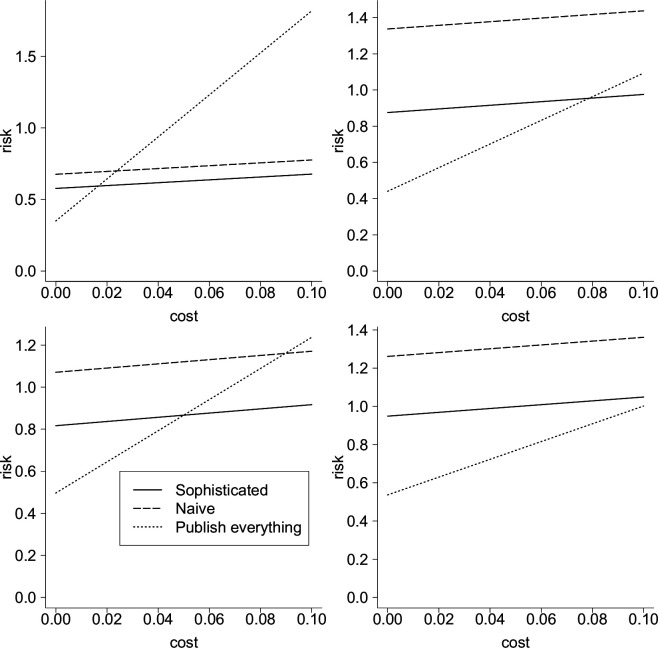
Risk as a function of c, showing scenario 1 as a solid line, 2 as a dashed line and 3 as a dotted line. The parameters are μ=0, σ=1, τ=2 (top left), μ=0, σ=2, τ=1 (top right), μ=2, σ=1, τ=2 (bottom left), μ=2, σ=2, τ=1 (bottom right). In all cases, $k=z_{\alpha /2}\cdot \tau$ with α=0.05. See [48] for code.

To this end, we numerically estimate the risk for a range of parameter values using R [47]. A selection of representative results is presented in figure 1. For various parameter values, we show the risk for the well-informed scientist (solid line), the naive scientist (dashed line) and when everything is published (dotted line).

As expected, publishing everything is best when c=0, but increases more quickly than the two publication bias scenarios. Because the naive scientist always does worse than the well-informed scientist, the range of values of c for which the naive scientist prefers publishing everything is larger than the range of values of c for which the well-informed scientist prefers publishing everything. Since we have argued that the risk for go-betweens is especially important and go-betweens should be expected to act more like naive scientists, the former comparison is the more relevant one. In the two left-hand panels of figure 1, we can see the dashed and dotted lines cross, indicating the value of c above which publication bias yields a lower risk than publishing everything. In the right-hand panels, publishing everything is better for all shown values (0≤c≤0.1).

Are the values shown for c ‘realistic’? This will vary from case to case. However, note that if c is too high relative to the epistemic or social cost of making a poor decision regarding the scientific problem at hand, then it would be optimal for the scientist to see no data at all and make an immediate decision (see [49], §5 and especially fig. 1). Since we are comparing three scenarios that each involve looking at at least some data, it seems reasonable to assume that we are not in a case where looking at any data would be irrational; we might say that any problem of genuine scientific interest has to be of enough social or epistemic importance to justify collecting and evaluating evidence about it. In figure 1, the social or epistemic cost of a poor decision is indicated by looking at the risk when c=0, as this isolates the squared error loss from the scientist’s estimate of θ from the opportunity cost of attending to the data. We see that in all four cases, the social or epistemic cost is no greater than 1 (at least for the well-informed scientist or in the publish everything scenario). Therefore, when c=0.1, the social or epistemic cost of a poor decision is no more than 10 times as large as the cost of attending to a single dataset (this corresponds to *β*/*c* ≤ 10 in fig. 1 of [49]). If c were much higher, the scientist would prefer to see no data at all. So the range of values shown for c seems realistic for questions of genuine scientific interest.

With these numerical simulations taken into account, we see that while there are some scenarios where publication bias leads scientists to be better off than publishing everything, this will not hold for cases that better resemble the actual problems scientists face. When we consider cases with realistic levels of opportunity cost, publishing everything will be better. This holds especially for the naive scientists or go-betweens. Since we have argued that the mediate aim of science is best served by tending to the needs of the latter group, this undermines the argument against anti-publication bias policy presented in §3.

## 6. Conclusion

We have argued that a very well-informed scientist may not believe that she stands to gain much from anti-publication bias policy. However, many more people need to make use of scientific information than just well-informed scientists. These more naive reasoners, often go-betweens translating scientific results into policy or public edification, will pay a much higher epistemic cost for publication bias. As such, our arguments ultimately support the case for anti-publication bias policy.

In more detail, a community that exhibits publication bias will render go-betweens statistically inconsistent. They should (typically) be willing to pay to reduce this publication bias, given how much error it is generating for them. However, the social benefits of science often depend on such people understanding and applying its results well. There is hence a case to be made for even well-informed scientists being compelled to comply with costly anti-publication bias strategies, such as mandatory preregistration or publishing on the basis of accepted experimental designs.

That said, we emphasize that we are not yet in a position to conclude anything stronger than that there is a case to be made. Our point in this paper is dialectical: we have sought to undermine arguments against anti-publication bias policy by showing that even if quite favourable assumptions are granted, the role of go-betweens still suggests a need for anti-publication bias policy. Before one actually implemented such a policy one would need to make an all-things-considered judgement that it was worth it. This would require considering many more factors than just those featured in the idealized models presented here.

First, one would need to think about the potential side effects of different forms of anti-publication bias policy, and their potential for introducing new biases into scientific social structures. For instance, if prepublication agreements were easier to obtain for scientists who have already established a reputation for interesting results, then this could make getting grants or tenure harder for less prestigious or more junior scientists, thus amplifying prestige bias in science. Prestige bias plausibly harms the mediate aims of science, for instance by making it harder to explore fresh new ideas associated with more junior members of the profession.

Second, there are further aspects of publication bias to consider. While in this paper we have focused on the effects of publication bias on readers of scientific journals, a full evaluation should also take into account its effects on authors. What are the financial and opportunity costs of having scientists do studies that they cannot publish? and how are those costs distributed? This point again highlights that some individual scientists or go-betweens might be affected very differently from others by whether and how we choose to intervene on publication bias.

Third, anti-publication bias policy could end up affecting what sort of work scientists do. For instance, it might force them to write up papers they would not otherwise have written, and certain styles of scientific paper or modes of analysis may become more or less popular as a result of this change in how scientists use their time.

The opportunity costs and epistemic effects of such trade-offs have to be modelled or investigated before one makes any recommendations, and we may decide we do not want to meddle in the social structure of science. After all, we could always try to target the norms or incentive structures among the go-between agents more directly. This, however, would similarly require engaging in a detailed analysis of their socio-epistemic situation.

Building towards such an all-things-considered evaluation will be the task of much future work. For now we reiterate we hope that as this discussion continues, the mediate aims of science are kept firmly in mind. We do not pursue science for the sake of producing better informed scientists. If it is worth it to implement anti-publication bias policy, it must be because the all-things-considered benefits accrue to those whose perspectives most matter for the mediate aims of science. Striving for an unbiased publication record is worth it not for the sake of what it tells scientists, but for what it tells the rest of us.

## Data Availability

A proof of theorem 1 is available as electronic supplementary material to this article (an additional external link is here: [27]. All simulation code used is provided at [48]. Supplementary material is available online [50].

## Footnotes

^1^
Why do we assume a Bayesian framework? This is mainly for rhetorical clarity: all the points we will make could also be made in a sufficiently sophisticated frequentist framework. However, since the phenomenon of publication bias is connected to the naive application of frequentist ideas, presenting our points in frequentist terms would mean analysing a potentially confusing mixture of naive and sophisticated frequentist methods. We emphasize, therefore, that we do not claim here that Bayesian statistics is superior to frequentist statistics on this point. The points made here from the Bayesian perspective could be recovered by a sufficiently sophisticated frequentist (in particular, one that accounts for publication bias in interpreting published data).

^2^
In the case of a two-tailed *Z*-test, the threshold k is determined by the significance level α according to the formula $k=z_{\alpha /2}\cdot \tau$. For example, if α=0.05 then $z_{\alpha /2}\approx 1.96$ and if α=0.01 then $z_{\alpha /2}\approx 2.58$. If instead the null hypothesis were either θ≤0 or θ≥0 then a one-tailed test would be used instead and the data would be considered statistically significant if $X>\tau \cdot z_{\alpha }$ or $X<-\tau \cdot z_{\alpha }$, respectively.

^3^
Recall that in Bayesian statistics, and Bayesian epistemology more generally, we assume that scientists' beliefs can be modelled using probabilities. A scientist's beliefs about the value of an unknown quantity (such as the parameter θ) are captured in a probability distribution (known as the *prior*) indicating how likely she thinks it is that the true value of the quantity might be this or that. Crucially, the Bayesian methodology assumes also that when new evidence comes in, the scientist updates her beliefs by conditionalizing on the new information. Bayes’ law offers a formula for this: the updated beliefs are proportional to the prior density multiplied by the likelihood (i.e. the probability density function conditional on the value of θ). The new beliefs are known as the *posterior*.

^4^
We can specify the likelihood function for Y explicitly: $f_{Y,\theta }(y)=\left\{ \begin{array}{ll} \frac{f_{X,\theta }(y)}{{\mathrm{Pr}}_{\theta }(|X|>k)}=\frac{1}{\tau }\frac{\phi \left(\frac{y-\theta }{\tau }\right)}{\Phi \left(\frac{-k-\theta }{\tau }\right)+1-\Phi \left(\frac{k-\theta }{\tau }\right)} & \text{ if }|y|>k\\ 0 & \text{ otherwise } \end{array}\right.$where ϕ and Φ are respectively the probability density function and cumulative density function of the standard normal distribution.

^5^
The naively calculated posterior density is $\pi_{X}(\theta |y)=\frac{1}{s}\phi \left(\frac{\theta -m}{s}\right)$. The posterior density calculated with the correct likelihood function for Y is given (up to a proportionality constant) by

$$\pi_{Y}(\theta |y)\propto \frac{1}{s}\frac{\phi \left(\frac{\theta -m}{s}\right)}{\Phi \left(\frac{-k-\theta }{\tau }\right)+1-\Phi \left(\frac{k-\theta }{\tau }\right)}=\frac{\pi_{X}(\theta |y)}{\Phi \left(\frac{-k-\theta }{\tau }\right)+1-\Phi \left(\frac{k-\theta }{\tau }\right)}.$$

The denominator is minimized at θ=0 and monotonically increases to one as θ→±∞. Thus, relative to $\pi_{X}(\theta |y)$, $\pi_{Y}(\theta |y)$ puts higher density near zero and lower density away from zero. If n independent draws from Y are observed a version of the above still holds, except the denominator is raised to the power n:

$$\pi_{Y_{1},\ldots ,Y_{n}}(\theta |y_{1},\ldots y_{n})\propto \frac{\pi_{X_{1},\ldots ,X_{n}}(\theta |y_{1},\ldots ,y_{n})}{{\left(\Phi \left(\frac{-k-\theta }{\tau }\right)+1-\Phi \left(\frac{k-\theta }{\tau }\right)\right)}^{n}}.$$

^6^
How is this possible? Given a parametric model of the underlying data-generating process and a parametric model of the possible ways publication bias and p-hacking distort what is published, the distribution of the sample means that is actually published will be different depending on the presence and extent of publication bias and p-hacking actually operative (see [17], fig.1 for a nice illustration of this). As the published literature grows, the scientist starts to see the shape of this distribution, allowing her to infer both the presence and extent of publication bias and the parameters of the underlying process. The Bernstein-von Mises theorem shows that this works in general.

^7^
Under weaker assumptions than the Bernstein-von Mises theorem, Doob's consistency theorem ([20], for a modern presentation see [15], theorem 10.10) yields consistency of the posterior for *almost all* possible values of the parameter, i.e. inconsistency arises only for a set of parameter values that has prior probability zero. The weaker assumptions mean that Doob's theorem applies widely, beyond relatively simple parametric models. However, the null set where inconsistency arises can be problematic, especially in problems with infinite-dimensional parameter spaces. See [21] for discussion.

^8^
An alternative approach eschews large sample results altogether. Suppose the scientist faces some decision problem. Fix the scientist's prior and the data she has access to. Then it is a theorem of statistical decision theory that if the scientist (correctly) works out her posterior and bases her decision on that posterior, the resulting decision will be optimal (in the sense of minimizing risk or maximizing expected utility) relative to the information available to her ([14], theorem 12.7). Insofar as the naive approach produces an incorrect posterior it will lead to worse decisions by the scientist's own lights. We return to this point in §5.

^9^
The prior might be normally distributed, as we assumed in §2, but this is not essential. Our result below applies to any prior, as long as it does not put positive probability mass on the hypothesis θ=0 and does not depend on the threshold of statistical significance k.

^10^
We emphasize that we assume a context in which whether θ is positive or negative is the only way in which the value of θ matters, i.e. the precise effect size is irrelevant. This is a slightly unusual context but we see no reason to think this could never happen in science. Since all we aim to show is that a well-informed scientist *sometimes* prefers to be in the first scenario, this suffices for our purposes. We consider a more typical decision context in §5.

^11^
Apart from its implications for the import of publication bias, our example may also be interpreted as a challenge to [23]. While we do not dispute the theorem that a Bayesian decision-maker should always use free information when offered, we have emphasized that information is never free. We think Good [23] would agree, as he consistently writes ‘negligible cost’ rather than ‘free'. However, for any putative meaning of ‘negligible’ we have constructed a case where the information is not worth having even at negligible cost. This significantly reduces the practical import of Good [23], as it implies one can never treat the cost of information as negligible without actually analysing the relevant decision problem(s).

^12^
As noted above, if she starts with a sufficiently flexible prior, such an agent can bootstrap herself to more complete knowledge of the nature and extent of publication bias and related distorting influences such as p-hacking [17–19]. Though in the short run such an agent would be quite unsure what is going on, and would thus stand to benefit from anti-publication bias policy. Agents who are mistaken rather than unsure, i.e. those whose prior rules out the particular form of publication bias actually present in the data they are learning from, will generally not be able to engage in this kind of bootstrapping.

^13^
Moreover, it is demonstrably worse in all decision problems, as we noted in footnote 8. We illustrate this in some numerical examples below.
